# Role of Thoracic Ultrasound in the Management of Parapneumonic Effusion According to the Proposed Algorithm/Classification in Pediatric Pneumonia: A Single Center Study

**DOI:** 10.1002/hsr2.72750

**Published:** 2026-07-14

**Authors:** Seyed Ali Nourbakhsh Kumeleh, Mehran Hiradfar, Majid Sezavar, Mohammad Hassan Aelami, Mohammad Saeid Sasan, Abdolkarim Hamedi, Seyed Javad Sayedi, Ali Khakshour, Reza Shojaeian, Ahmad Mohammadipour, Hamid Reza Kianifar, Masoud Mahdavi Rashed, Seyed Ali Alamdaran

**Affiliations:** ^1^ Student Research Committee, Faculty of Medicine Mashhad University of Medical Sciences Mashhad Iran; ^2^ Department of Pediatric Surgery Mashhad University of Medical Sciences Iran; ^3^ Department of Pediatrics, Pediatric Intensive Care Division, Faculty of Medicine Mashhad University of Medical Sciences Mashhad Iran; ^4^ Department of Pediatric Infectious Disease, Faculty of Medicine Mashhad University of Medical Sciences Mashhad Iran; ^5^ Mashhad University of Medical Sciences Mashhad Iran; ^6^ Department of Pediatrics, Faculty of Medicine Mashhad University of Medical Sciences Mashhad Iran; ^7^ Faculty of Medicine Mashhad University of Medical Sciences Mashhad Iran; ^8^ Radiology Department, Faculty of Medicine Mashhad University of Medical Sciences Mashhad Iran

**Keywords:** clinical algorithm, parapneumonic effusion, pediatric pneumonia, pleural effusion, thoracic ultrasound, ultrasound‐guided management

## Abstract

**Background and Objective:**

Parapneumonic effusion refers to any pleural effusion secondary to pneumonia. Thoracic ultrasound is the imaging modality of choice for evaluating pleural effusion. This study aimed to assess the role of thoracic ultrasound, guided by a proposed algorithm and classification system, in the management of parapneumonic effusion and the prevention of related complications in children with pneumonia.

**Methods:**

Between 2020 and 2022, children presenting with clinical symptoms of pneumonia were evaluated by pediatricians at Akbar Children's Hospital in Mashhad. Patients with chest X‐rays suggestive of parapneumonic effusion were enrolled. Management and treatment were guided by a specialist‐designed algorithm. Patients were stratified into two groups: those with underlying disease and those without. Each group was analyzed separately.

**Results:**

Among the 105 enrolled children, 48 had underlying conditions and 57 did not. Ultrasound revealed pulmonary consolidation in all cases. The most frequent effusion stage in both groups was < 10 mm (43.9% in patients without underlying disease and 58.3% in those with underlying disease). None of the patients with stage II or III effusion who received algorithm‐based treatment progressed to a higher stage during follow‐up. A significant increase in complications was observed among patients initially admitted with advanced effusion stages (*p* = 0.002).

**Conclusion:**

The findings suggest that implementing the proposed ultrasound‐based algorithm/classification system is effective tools in managing parapneumonic effusion and preventing complications in pediatric pneumonia.

## 1 Introduction

Pneumonia remains one of the most prevalent respiratory diseases in children and is a leading cause of morbidity and mortality among those under 5 years of age worldwide [1, 2, 3]. Although diagnosis is primarily based on clinical findings, imaging modalities are often required to support assessment and guide management [4]. Chest X‐ray (CXR) is typically the first‐line imaging tool; however, its utility is limited by several factors, including exposure to ionizing radiation, low negative predictive value, limited sensitivity in detecting early complications, and interobserver variability in interpretation [5]. While computed tomography (CT) and magnetic resonance imaging (MRI) have been proposed as complementary modalities, their use is constrained by high radiation exposure and cost, respectively [6].

Thoracic ultrasound has emerged as a valuable alternative for evaluating pneumonia in both pediatric and adult populations [7, 8]. Unlike CT and MRI, ultrasound is a rapid, non‐invasive, and radiation‐free technique that can be performed at the bedside [9]. Additional advantages include its widespread availability, short examination time, affordability, repeatability, and utility in monitoring treatment response [10]. Importantly, thoracic ultrasound is considered the imaging modality of choice for assessing parapneumonic effusion. It offers superior sensitivity compared to CXR in detecting small pleural effusions and enables quantification of effusion volume, identification of fibrinous septations, and differentiation between pleural effusion, lung consolidation, peripheral lung abscess, and empyema [11, 12, 13, 14, 15]. These capabilities are critical for predicting complications associated with pediatric pneumonia [16]. Furthermore, Doppler ultrasound may facilitate early detection of necrotic changes [17].

This study aims to evaluate the role of early thoracic ultrasound, guided by a proposed algorithm and classification system, in the management of parapneumonic effusion and the prevention of related complications in children with pneumonia.

## 2 Methods

This single‐center study was conducted at Akbar Children's Hospital, affiliated with Mashhad University of Medical Sciences, between 2020 and 2022. Pediatric patients with clinical symptoms of pneumonia and chest X‐ray (CXR) findings suggestive of parapneumonic effusion were enrolled. Demographic data including age, sex, and presence of underlying disease were recorded.

Children presenting with symptoms such as fever, cough, lethargy, anorexia, nausea, vomiting, and other respiratory complaints were referred for CXR imaging. Patients with radiographic signs of parapneumonic effusion—including lower zone opacification, blunting of the costophrenic angle, or visible pleural fluid—were eligible for inclusion.

All eligible patients underwent thoracic ultrasound to confirm the presence and characteristics of parapneumonic effusion and to assess pneumonia‐related complications. Ultrasound examinations were performed by experienced pediatric radiologists using a GE E6 ultrasound system equipped with linear and convex probes. For optimal visualization of free pleural effusion, patients were positioned in the lateral decubitus posture at the bedside. Effusions were classified into four stages based on sonographic features, including thickness, loculation, septation, and pleural thickening. A scoring system was applied, assigning two points for effusions > 1 cm, two points for multiple septa, and four points for pleural thickening—analogous to other RADS (Reporting and Data System) frameworks. Management options included clinical follow‐up, chest tube placement, video‐assisted thoracoscopic surgery (VATS), fibrinolytic therapy, and decortication.

**Figure 1:**
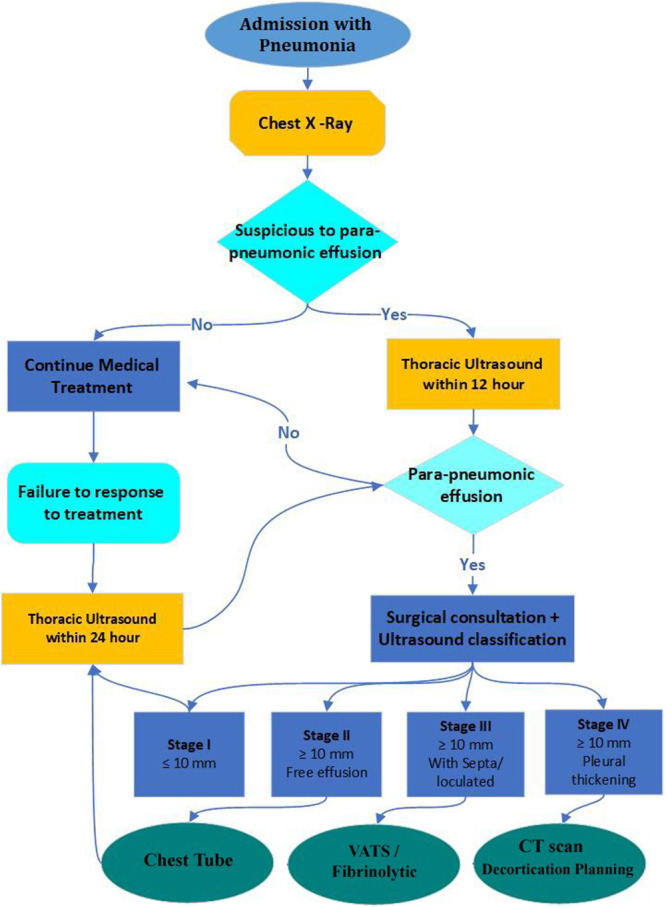
Proposed algorithm for ultrasound ‐ guided management of parapneumonic effusion in children.

**Figure 2:**
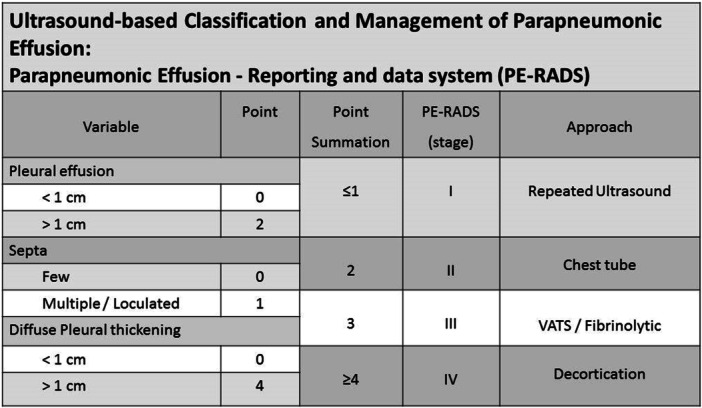
Proposed ultrasound ‐ based classification and management of parapneumonic effusion (Parapneumonic Effusion – Reporting and Data System).

Patient management was guided by a multidisciplinary algorithm and classification system (Figure 1), developed collaboratively by radiologists, pediatric surgeons, pulmonologists, infectious disease specialists, and intensivists. (Figure 2) These figures illustrate the ultrasound‐based reporting and decision‐making framework for parapneumonic effusion. Patients who declined recommended interventions or were managed outside the proposed algorithm were excluded from the study.

All patients were systematically evaluated for pneumonia‐related complications, duration of hospitalization, need for intensive care unit (ICU) admission, and surgical interventions throughout their hospital stay. Findings were documented in detailed clinical reports.

Patients were stratified into two groups based on the presence or absence of underlying disease. Those with debilitating conditions—including cerebral palsy, congenital or acquired cardiac disease, immunodeficiency, Down syndrome, epilepsy, and neuromuscular disorders—were classified as having underlying disease. Each group was analyzed separately.

Following completion of standardized checklists, data were entered into SPSS version 24 for statistical analysis. Descriptive statistics were presented using tables, figures, and measures of central tendency and dispersion. Data normality was assessed using the Kolmogorov–Smirnov test. For quantitative variables, the independent samples *t*‐test was applied to normally distributed data, while the Mann–Whitney test was used for non‐normal distributions. Associations between qualitative variables were examined using the Chi‐square test and Fisher's exact test. Correlation analyses were performed using Pearson's test for normally distributed data and Spearman's test for non‐normal data. A *p* < 0.05 was considered statistically significant.

This study was approved by the Medical Ethics Committee of Mashhad University of Medical Sciences (IR.MUMS.IRH.REC.1402.063). As all participants were under 18 years of age, written informed consent was obtained from their parents, legal guardians, or authorized representatives.

## 3 Result

A total of 105 children diagnosed with pneumonia were enrolled and underwent chest X‐ray (CXR) and thoracic ultrasound evaluation according to the proposed algorithm and classification system. Out of these, 55 patients were male and 50 were female. The mean age of patients without underlying disease was 5.52 ± 5.07 years, whereas the mean age of those with underlying disease was 6.54 ± 5.46 years.

Pneumonic involvement was observed on the right side in 44 patients, on the left side in 29 patients, and bilaterally in 32 patients. Forty‐eight patients (45.7%) had documented underlying diseases, while 57 patients (54.3%) had no known comorbidities. The most prevalent underlying condition was cerebral palsy (CP), identified in 11 patients (10.5%). Other comorbidities included congenital or acquired cardiac disease, immunodeficiency, Down syndrome, epilepsy, and neuromuscular disorders.

Thoracic ultrasound revealed pulmonary consolidation in all patients (105/105; 100%). Air bronchograms were identified in all patients with underlying disease (48/48; 100%) and in 54 patients (94.7%) without underlying disease.

Table 1 summarizes the ultrasound‐based staging, frequency, and associated complications of parapneumonic effusion across both patient groups. The most common finding in both cohorts was a pleural effusion measuring less than 10 mm in thickness observed in 43.9% of patients without underlying disease and 58.3% of those with underlying disease.

**Table 1:** Stage, number, and complications in 105 patients with parapneumonic effusion.

Thickness of effusion	Stage	Total	Without underlying disease		With underlying disease	
		Number (%)	Number (%)	Complications	Number (%)	Complications
Less than 10 mm	I	53 (50.5)	25 (43.9)	5 (20)	28 (58.3)	1 (2)
More than 10 mm	II	17 (16)	5 (8.8)	0	12 (25)	0
	III	9 (8.5)	7 (12.3)	3 (42.9)	2 (4.2)	0
	IV	26 (25)	20 (35.1)	14 (70)	6 (12.5)	0
Total		105 (100)	57 (54.3)	22 (21)	48 (45.7)	1 (0.9)

In the cohort of children without underlying disease, parapneumonic effusion–related complications included empyema (63.64%), multisystem inflammatory syndrome (MIS) (22.73%), necrotizing pneumonia (9.09%), and lung abscess (4.55%).

Correlation analyses using Pearson's test were conducted to assess associations between clinical outcomes (recovered vs. deceased) and categorical variables. Among patients without underlying disease, no statistically significant associations were found for gender (*p* = 0.631), side of lung involvement (*p* = 0.647), effusion stage (*p* = 0.576), complication occurrence (*p* = 0.635), or type of management (*p* = 0.935). The corresponding odds ratios (OR) were all close to 1.0 with 95% confidence intervals (CI) crossing 1, further supporting the absence of a meaningful association.

In contrast, among patients with underlying disease, bilateral lung involvement was significantly associated with mortality, being more frequent in deceased patients (69.2%) than in those who recovered (*p* = 0.024). This represents a large and significant effect, with patients having bilateral involvement being over 8 times more likely to die (OR = 8.44, 95% CI [1.33, 53.55]). No other variables in this group, including gender (*p* = 0.329), effusion stage (*p* = 0.425), and type of management (*p* = 0.151), showed a significant association with outcome, with effect sizes confirming this lack of association.

Notably, no patients with stage I or II effusion who received algorithm‐based treatment progressed to a higher stage during follow‐up. However, a significant increase in complication rates was observed among patients initially admitted with advanced stages (*p* = 0.002). The odds of having a complication were markedly higher in advanced stages (III/IV) compared to early stages (I/II) (OR = 10.80, 95% CI [2.44, 47.78]).

Management decisions, including follow‐up, surgical referral, and procedural interventions, were guided by the proposed algorithm. The distribution of these strategies and the corresponding clinical outcomes are presented in Table 2. Follow‐up was the most frequently employed management approach, accounting for 61% of cases.

**Table 2:** Management and outcome of 105 patients with parapneumonic effusion.

Management	Total	Without underlying disease	With underlying disease
	Number (%)	Number (%)	Number (%)
Follow up	64 (61)	26 (45.6)	37 (77)
Decortication	22 (21)	18 (31.6)	4 (8)
Chest tube	9 (8.5)	4 (7)	5 (10)
Lobectomy	4 (3.8)	4 (7)	0
VATS	4 (3.8)	4 (7)	0
Bronchoscopy	1 (0.9)	1 (1.8)	0
Lack of consent to treatment	4 (4)	1 (2)	3 (6)
Recovered	84 (80)	52 (91)	32 (67)
Dead	17 (16)	4 (7)	13 (27)
Total	105	57	48

A small subset of patients (*n* = 4, 8%) declined the recommended treatment and were managed outside the proposed algorithm; these cases were excluded from the final analysis of treatment efficacy.

Data normality was assessed using the Kolmogorov‐Smirnov test. The Mann‐Whitney test was applied for non‐normally distributed data to examine the relationships between clinical outcomes and quantitative variables (patient age, ward stay duration, and ICU stay duration). In the general ward, the stay was 18.37 ± 10.77 days for patients without underlying disease and 20.42 ± 16.08 days for those with underlying conditions. In the intensive care unit (ICU), the average length of stay was 8.16 ± 9.73 days for patients without underlying disease and 13.75 ± 14.17 days for those with underlying disease.

Among patients without underlying disease, a longer ICU stay was significantly associated with mortality (*p* = 0.009). The effect size for this difference was large (1.24), indicating that ICU stay was, on average, over one standard deviation longer in deceased patients. No significant associations were found with age (*p* = 0.729) or ward stay duration (*p* = 0.227). For patients with underlying disease, no significant associations were observed for age (*p* = 0.062), ward stay (*p* = 0.727), or ICU stay (*p* = 0.458), with corresponding small effect sizes (< 0.35 for all).

Final clinical outcomes for all 105 children were assessed. In the group without underlying disease, 52 (91.2%) recovered, and 4 (7.0%) died. In the group with underlying disease, 32 (66.7%) recovered, and 13 (27.1%) died. The presence of an underlying disease was a strong risk factor for mortality, with affected patients having over 5 times the odds of dying compared to those without underlying diseases (OR = 5.28, 95% CI [1.62, 17.19]). The risk ratio was 3.87, meaning mortality was nearly 4 times more frequent in the group with comorbidities. In this latter group, mortality was primarily associated with the presence of underlying conditions and bilateral lung involvement.

## 4 Discussion

Parapneumonic effusion refers to any pleural effusion secondary to bacterial or viral pneumonia or lung abscess. While most children with community‐acquired pneumonia recover uneventfully, a subset may develop local or systemic complications. Local complications include parapneumonic effusion, empyema, necrotizing pneumonia, and lung abscess [18]. The incidence of parapneumonic effusion in pediatric pneumonia ranges from 5% to 40%, depending on disease severity and diagnostic criteria.

Thoracic ultrasound has proven highly effective in identifying pneumonia‐related complications, particularly empyema, necrotizing pneumonia, and lung abscess—among the most common sequelae in pediatric patients [19]. Its diagnostic validity spans across all pediatric age groups [7, 8]. Multiple studies, including our own, have demonstrated that ultrasound is a reliable and precise alternative to chest radiography (CXR) for evaluating pneumonia in children [20, 21, 22, 23, 24, 25]. One study reported a higher detection rate of lung consolidation using thoracic ultrasound compared to CXR [15]. Another comparative study found that thoracic ultrasound outperformed both CXR and CT in guiding treatment decisions for children with parapneumonic effusion [13]. Consistent with these findings, our study revealed that all patients with parapneumonic effusion also exhibited pulmonary consolidation on ultrasound.

Despite its clinical significance, the management of parapneumonic effusion in children remains controversial. Currently, there is no universally accepted guideline or consensus on standardized treatment protocols for pediatric parapneumonic effusion [26, 27, 28]. As a result, therapeutic approaches are often center‐dependent and vary based on institutional expertise and available resources. In this study, we evaluated children diagnosed with parapneumonic effusion who underwent chest radiography and thoracic ultrasound, and were managed according to a multidisciplinary algorithm and classification system specifically designed for this purpose.

Due to the referral nature of our medical center, some patients with advanced‐stage parapneumonic effusion (stage III or IV) did not undergo initial ultrasound evaluation. This was primarily attributed to prior assessment at other facilities or delays in referral. Notably, patients with stage II or III effusion who received algorithm‐based treatment did not progress to higher stages during follow‐up. These findings underscore the importance of timely thoracic ultrasound in children with pneumonia, enabling early detection and intervention that may prevent disease progression and reduce complication rates. Early diagnosis of pleural effusion is particularly valuable, as it often allows for less invasive management and may obviate the need for surgical procedures such as decortication or lobectomy.

Our study demonstrated a significant correlation between the stage of parapneumonic effusion and the incidence of complications, with higher complication rates observed in patients presenting at advanced stages (*p* = 0.002). Recovery rates were notably higher among children without underlying disease compared to those with comorbidities. In patients without underlying disease, prolonged ICU hospitalization was associated with increased mortality, whereas in patients with underlying conditions, bilateral lung involvement was the primary contributor to poor outcomes. Importantly, management guided by the proposed ultrasound‐based algorithm led to clinical improvement and hospital discharge in both patient groups, underscoring its potential utility across diverse clinical profiles.

The ultrasound‐based classification and management algorithm used in this study was developed through multidisciplinary collaboration involving pediatric pulmonologists, infectious disease specialists, intensivists, radiologists, and pediatric surgeons. A key strength of this algorithm lies in its integrative design, which facilitates coordinated decision‐making while enabling timely surgical consultation when necessary. By promoting structured yet flexible management of parapneumonic effusion, the algorithm ensures prompt diagnosis and appropriate intervention—while preserving the clinical autonomy of the primary physician.

Our findings suggest that implementing the proposed ultrasound‐based algorithm for managing parapneumonic effusion can facilitate recovery while minimizing patient burden and long‐term risks—particularly radiation exposure during early childhood. Although no universally standardized treatment exists for parapneumonic effusion, structured algorithms such as the one presented here can reduce unnecessary diagnostic procedures and enable timely, targeted interventions that help prevent disease‐related complications. These results underscore the urgent need for evidence‐based guidelines to standardize the management of parapneumonic effusion and reduce reliance on invasive or time‐consuming procedures, including pleural tapping, microbiological cultures, and CT imaging.

The integration of ultrasound into the proposed management algorithm offers numerous clinical advantages. Ultrasound demonstrates exceptional sensitivity in detecting even small pleural effusions, facilitates early diagnosis of parapneumonic effusion, and enables non‐invasive monitoring throughout the course of treatment. Its repeatability at any stage of care allows for dynamic assessment without the need for sedation or exposure to ionizing radiation. Moreover, the immediate availability of ultrasound results supports timely clinical decision‐making when combined with physical examination and laboratory findings. In our study, ultrasound proved to be a valuable tool not only for diagnosing and managing parapneumonic effusion, but also for the early detection and treatment of serious complications such as necrotizing pneumonia and lung abscess.

This study has several limitations. First, its single‐center design may introduce selection bias and limit the generalizability of findings. Multicenter studies are recommended to validate the proposed algorithm across diverse clinical settings. Second, the heterogeneity of underlying conditions—such as cerebral palsy, congenital heart disease, immunodeficiency, Down syndrome, and epilepsy—may contribute to comorbidity bias and influence clinical outcomes. Future research should aim to stratify patients more precisely and assess the algorithm's performance across specific subgroups. Further prospective studies are warranted to confirm the clinical utility and reproducibility of this ultrasound‐based classification system in the management of parapneumonic effusion in children.

## 5 Conclusion

This study demonstrated that the proposed ultrasound‐based algorithm and classification system for parapneumonic effusion (PE‐RADS) offers a valuable and effective framework for the diagnosis, treatment, and management of this complication in children. By enabling early identification of parapneumonic effusion and its associated complications, the algorithm supports timely and appropriate clinical decision‐making, reduces the risk of adverse outcomes, and facilitates faster recovery. Future studies should further investigate the role of ultrasound and similar structured approaches in monitoring high‐risk cases and assessing the impact of emerging therapies, such as fibrinolytics.

## Author Contributions

**Seyed Ali Nourbakhsh Kumeleh:** investigation, conceptualization, methodology, data curation, writing – original draft, formal analysis, writing – review and editing. **Mehran Hiradfar:** investigation, data curation. **Majid Sezavar:** investigation, data curation. **Mohammad Hassan Aelami:** investigation, data curation. **Mohammad Saeid Sasan:** investigation, data curation. **Abdolkarim Hamedi:** investigation, data curation. **Seyed Javad Sayedi:** investigation, data curation. **Ali Khakshour:** investigation, data curation. **Reza Shojaeian:** investigation, data curation, methodology. **Ahmad Mohammadipour:** investigation, data curation. **Hamid Reza Kianifar:** investigation, data curation. **Masoud Mahdavi Rashed:** investigation, methodology, data curation, writing – review and editing, writing – original draft, formal analysis. **Seyed Ali Alamdaran:** conceptualization, investigation, formal analysis, methodology, data curation, writing – original draft, writing – review and editing, project administration, supervision.

## Funding

The authors have nothing to report.

## Ethics Statement

This study was conducted in accordance with scientific and ethical standards. All procedures were performed in compliance with relevant guidelines and regulations. The study protocol was approved by the Medical Ethics Committee of Mashhad University of Medical Sciences.

## Consent

The authors have nothing to report.

## Conflicts of Interest

The authors declare no conflicts of interest.

## Transparency Statement

The Seyed Ali Alamdaran affirms that this manuscript is an honest, accurate, and transparent account of the study being reported; that no important aspects of the study have been omitted; and that any discrepancies from the study as planned (and, if relevant, registered) have been explained.

## Data Availability

The data that support the findings of this study are available on request from the corresponding author. The data are not publicly available due to privacy or ethical restrictions.
